# Equity Must Accompany Economic Growth for Good Health

**DOI:** 10.1371/journal.pmed.1000426

**Published:** 2011-03-15

**Authors:** K. Srinath Reddy

**Affiliations:** Public Health Foundation of India, Vasant Kunj, New Delhi, India

## Abstract

K. Srinath Reddy discusses a new research study by S. V. Subramanian and colleagues that found no strong evidence of recent economic growth in India being associated with a reduction in child undernutrition.

**Linked Research Article**

This Perspective discusses the following new study published in *PLoS Medicine*:

Subramanyam MA, Kawachi I, Berkman LF, Subramanian SV (2011) Is Economic Growth Associated with Reduction in Child Undernutrition in India? PLoS Med 8(3): e1000424. doi:10.1371/journal.pmed.1000424

An analysis of cross-sectional data from repeated household surveys in India, combined with data on economic growth, fails to find strong evidence that recent economic growth in India is associated with a reduction in child undernutrition.

India's rapid economic growth, after the initiation of market-oriented reforms two decades ago, has been the subject of considerable international attention. However, this accelerated economic growth has not been matched by marked improvements in many of the key indicators of health. Not only are the rates of infant and maternal mortality lagging behind the Millennium Development Goals, but the level of undernutrition among children is appallingly high. Even if there is a lag time for improvements in the economic status of countries to be reflected in better health indicators at the population level, child nutrition should be one of the earliest and highly sensitive indicators of economic growth favourably impacting health. Yet, this has not happened in India, where average calorie intake has declined over the last 25 years [1].

In *PLoS Medicine* this month, Subramanyam et al. [2] explored this paradox through multi-level modeling of cross-sectional data from three national surveys conducted during this period of economic growth at 6- to 7-year intervals. With per capita income at the state level as the exposure variable, they examined the association with different measures of undernutrition across several states in India. They did not observe any association between state economic growth with the risk of children being underweight, stunted, or wasted. Adjustment for several demographic and socioeconomic covariates did not alter this null effect.

The relationship of economic growth with health has earlier been studied with other indicators. In general, life expectancy increases and infant mortality decreases with rising national incomes. This effect is most marked in the early stages of economic growth, as evident from the Preston curves that are periodically developed to position countries along the line of relationship between per capita gross domestic product (GDP) and life expectancy [3]. It has also been well demonstrated that at equivalent levels of per capita GDP, countries with higher levels of economic and social equality within their societies fare better on most health indicators than countries where the equity gaps are wider. As Wilkinson and Pickett compellingly argue in their book *The Spirit Level* (2010), poor health is not merely the result of poverty but also of inequality, which manifests in many dimensions [4]. In unequal societies, it is not merely the poor who suffer from sub-optimal health, as other sections of society also are less well than their social class peers in more equal societies.

The pattern of India's economic growth over the last two decades has accentuated income inequality, with a large segment of the society not benefiting from it. The recent measure of persons living below the poverty level, even by official estimates, is 37.2% of the population. Those just above that level and even the middle class are also highly vulnerable to food price inflation. As such, aggregate national or state indicators of economic growth do not reflect the real purchasing power of many at the bottom and middle of the population pyramid.

There is also a poverty of opportunities—for education, employment, and income generation. Caste, gender, and religion too are among the social determinants that impact opportunities for development and access to health services. Only recently has there been an attempt to guarantee employment for the rural poor at levels that support subsistence. The ability of families to provide appropriate child nutrition is thus dependent on several enabling factors.

The demographic and socioeconomic variables chosen by the authors, though pertinent, do not cover issues like the quality of governance, which varies widely across states and also with successive governments and state administrations within states. The surveys also do not account for undernutrition-related deaths in childhood. There is an unanswered question: do states where infant mortality is declining faster have more surviving low birth weight babies who carry their disadvantage into early childhood, thereby masking the overall benefits of economic growth on child health?

The Integrated Child Development Services (ICDS) program of the government has specifically aimed to improve child nutrition, but did not cover the very vulnerable age group of 0–3 years. Universal breast feeding up to at least 6 months of age and timely introduction of complementary feeding, which would have helped to reduce undernutrition in the very vital early years of child growth, were missing elements of this program [5]. The poor nutritional status of mothers (58% of pregnant women were anemic and 36% of the ever-married women, in the childbearing age group, were underweight in the 2005–2006 survey) as well as the low levels of child immunization with risk of childhood infections also contribute to undernutrition. Unsafe drinking water and poor sanitation, which predispose to diarrheal diseases and other infections, also contribute to child malnutrition.

The message that comes out clearly from Subramanyam et al.'s article is that developing countries like India should not assume that economic growth will automatically translate into improved child nutrition and health. Measures for enhancing equity through inclusive growth, action on social determinants of health, and specific programs for improved early life nutrition will be needed if undernourished children are not to become the face of an economically advancing India.

## Abbreviations

GDP: gross domestic product

## References

[1] Deaton A, Dreze J (2009). Food and nutrition in India: facts and interpretations.. Economic & Political Weekly.

[2] Subramanyam MA, Kawachi I, Berkman LF, Subramanian SV (2011). Is economic growth associated with reduction in child undernutrition in India?. PLoS Med.

[3] Deaton A (2003). Health, inequality, and economic development.. Journal of Economic Literature.

[4] Wilkinson R, Pickett K (2010). The spirit level..

[5] Paul VK, Sachdev HS, Mavalankar D, Ramachandran P, Sankar MJ (2011). Reproductive health, and child health and nutrition in India: meeting the challenge.. Lancet.

